# Oral Biofilm: Development Mechanism, Multidrug Resistance, and Their Effective Management with Novel Techniques

**DOI:** 10.5041/RMMJ.10428

**Published:** 2021-01-19

**Authors:** Shakti Rath, Sourav Chandra Bidyasagar Bal, Debasmita Dubey

**Affiliations:** 1 Associate Professor (Research), Central Research Laboratory, Siksha ‘O’ Anusandhan (Deemed to be University), Bhubaneswar, Odisha, India; 2 Assistant Professor (Public Health Dentistry), Institute of Dental Sciences, Siksha ‘O’ Anusandhan (Deemed to be University), Bhubaneswar, Odisha, India; 3 Post Doctoral Fellow, Centre of Excellence in Natural Products and Therapeutics, Department of Biotechnology, Sambalpur University, Sambalpur, Odisha, India

**Keywords:** Antibiotic resistance, clinical management, dental plaque, novel techniques, oral biofilms

## Abstract

Biofilms are formed by the congregation of one or more types of microorganisms that can grow on a firm surface. Dental plaque is one of the most commonly forming biofilms in the oral cavity and appears as a slimy layer on the surface of the teeth. In general, the formation is slow, but biofilms are very adaptive to the changing environment, and a mature biofilm can cause many health-related problems in humans. These biofilms remain unaffected by antibiotics as they do not allow the penetration of antibiotics. Moreover, the increased level of virulence and antibiotic resistance of microorganisms in the oral biofilm or dental plaque has made its clinical management a serious challenge worldwide. Chlorhexidine-like antimicrobial drugs have been partially effective in removing such organisms; however, the precise and continuous elimination of these microorganisms without disturbing the normal microbial flora of the oral cavity is still a challenge. This review paper focuses on the process of oral biofilm formation, related complications, development of drug-resistant bacteria in these biofilms, and their effective management by the use of different novel techniques, available from various published research and review articles.

## INTRODUCTION

Biofilm can be defined as a layer of both Gram-positive and Gram-negative bacteria, which adhere and grow on any biotic or abiotic firm surface, surrounded by a self-formed mucilaginous matrix.^1^,^2^ Biofilms are ubiquitous, and they can have serious ill health effects on humans. They also grow in or on inanimate surfaces like medical devices, dental implants, surgical sutures, catheters, and artificial joints.

One of the prime examples is oral biofilm or dental plaque, which is a common phenomenon in the oral cavity of all mammals. The natural dentition and dental prostheses, including dentures and implants, are substrates for biofilms. The consumption of carbohydrate-rich food leads to increased secretion of organic acids by the bacteria forming oral biofilm or dental plaque. Subsequently, as the bacterial colonization increases, there is an overproduction of matrix polysaccharide alginate, and the mucoid biofilm facilitates resistance to antibiotics and to the host’s immune response. An extracellular polymeric substance (EPS) is produced by the bacteria, which causes chronic infection, and its treatment becomes difficult. Under untreated conditions, biofilm demineralizes the enamel, causing dental caries.^1^,^2^

## BIOFILM FORMATION AND COMPOSITION

Biofilms were first formally defined in the mid-1980s; however, detailed knowledge on their mechanism of development was gained only recently.

Formation of oral biofilm consists of five basic stages:^3^–^5^

1. Entry of pathogenic microorganisms into the oral cavity.
2. Congregation of pathogenic and normal microflora, and salivary pellicle formation.
3. Marked increase of the biofilm-forming bacteria, formation of a mucilaginous layer and the beginning of dysbiosis, and the beginning of irreversible adhesion.
4. Exchange of drug-resistant genes between pathogenic bacteria and normal oral microflora, along with completion of dysbiosis.
5. Maturation of the biofilm and bacterial spread.

**Figure 1:**
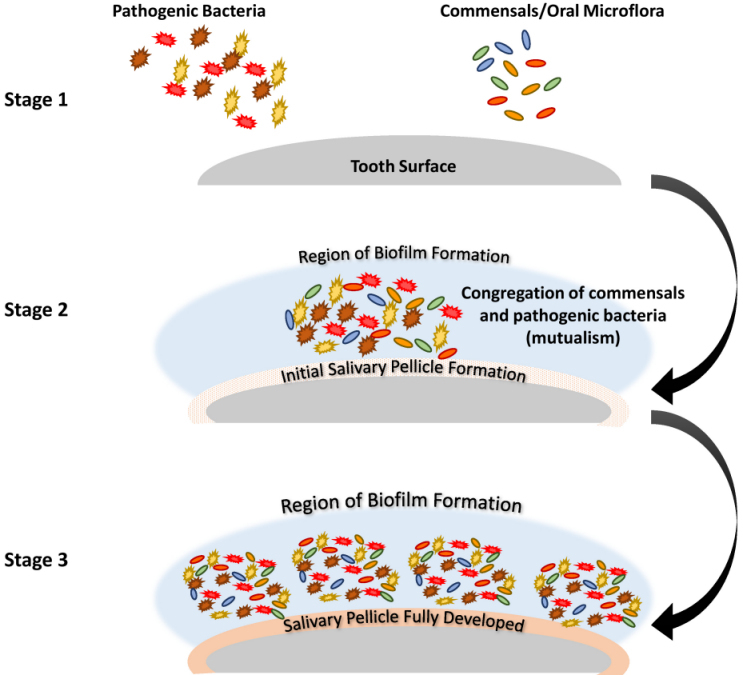
Schematic Description of Stages 1–3 of Biofilm Formation. **Stage 1:** Entry of multidrug-resistant pathogenic bacteria, commensals, and oral microflora. **Stage 2**: The salivary pellicle begins to form as the commensals and pathogenic bacteria congregate, laying the foundation for biofilm development. **Stage 3:** The bacteria rapidly multiply and begin to adhere, and dysbiosis begins.

**Figure 2:**
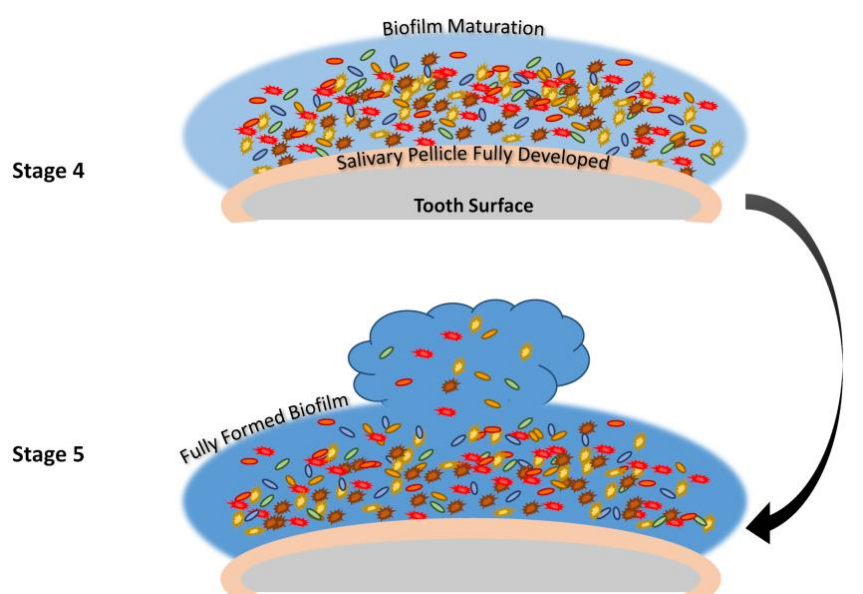
Schematic Description of Stages 4 and 5 of Biofilm Formation. **Stage 4:** The biofilm matures, there is an exchange of drug-resistant genes, and complete dysbiosis. **Stage 5:** With the full maturation of the biofilm, new multidrug-resistant bacteria are released into the oral cavity, spreading the oral biofilm.

Figure 1 depicts biofilm formation for stages 1–3, and Figure 2 depicts stages 4 and 5.

The formation of a biofilm begins with the entry of pathogenic microorganisms into the oral cavity. This process is still reversible at this stage, after which these bacteria will slowly start forming a layer over the teeth and produce a mucilaginous slimy covering which is irreversible. This covering consists of extracellular polysaccharides, structural proteins, cell debris, and nucleic acids—referred to as EPS—which protects the bacteria in the biofilm from mechanical stress and antimicrobial drugs.^3^–^5^ At the beginning, the matrix is formed by the extracellular DNA (eDNA), which is later succeeded by polysaccharides and structural proteins. Next, a marked increase of bacterial colonies takes place, and gene exchange occurs between some of the normal flora and the pathogenic bacteria, which can lead to the emergence of multidrug-resistant (MDR) or antibiotic-resistant (ABR) bacteria. The formation of biofilm occurs in a three-dimensional manner, and surface adhesion becomes irreversible. Subsequently, some bacteria move or spread out from the mucilaginous covering and initiate the formation of biofilm in other parts of the oral cavity (see also Figures 1 and 2).^3^–^5^

## BIOFILM-RELATED COMPLICATIONS

Biofilm acts as a predisposing factor for several oral infections, such as gingivitis, dental caries, periapical periodontitis, periodontitis, and peri-implantitis. Restorations, non-surgical or surgical periodontal therapies, root canal treatments, and dental implants are well established curative regimens that remove oral biofilm; however, these treatments are not always entirely successful at removing secondary biofilm infections. Biofilm management necessitates a very high rate of expenditure globally.^5^

Topically applied antibacterial drugs become diluted and are not effective for an extended duration due to the complexity of the oral cavity and rapid clearance by saliva. A mature biofilm requires a superior concentration of antimicrobial agents for its removal in comparison to bacteria in planktonic biofilms. When antimicrobial agents are used, only some cells are exposed, while some of the drug entrance is reduced by the EPS matrix of the biofilm, hence diffusion of drugs into the deeper layer of oral biofilm is prohibited.^4^ The planktonic biofilms differ from bacterial biofilm in gene expression, growth rate, translation, and transcription process, since they remain in a different type of microenvironment that has higher osmolarity, shortage of nutrients, and a higher cell density.^3^ The EPS matrix of oral biofilm helps in biofilm growth and maturation. Emergent properties include spatial and chemical heterogeneities, surface adhesion, competitive contact, and higher tolerance of antimicrobial agents.^1^ The bacteria residing inside the biofilm are protected from various environmental stresses, such as desiccation, attack from antimicrobial agents by the immune system, and ingestion of protozoa; therefore the oral biofilm structure makes for more complex bacterial communities in contrast to planktonic biofilm.^3^

The oral cavity provides ideal conditions for microbial growth and proliferation by providing a moist, warm, and nutritious environment. Microbial colonization of pathogenic biofilm occurs due to the complex dynamic interactions among the host, microorganisms, and diet. The biofilm produces acid at the tooth restoration margin, leading to secondary caries, which is also the reason for most restoration failures. Pulp infections are also seen after dental restorations. Even after root canal therapy, persistent biofilms inside the root canal system result in constant apical periodontitis and re-infection. Dental implants and periodontal tissues are affected by the biofilm, leading to peri-implantitis and periodontitis. Therefore, oral rehabilitation procedures depend on the capacity of dental materials to incorporate specific antibiotic strategies for controlling and eliminating these infections, which is the long-term clinical accomplishment. A critical assessment of recently published, innovative antimicrobial strategies for managing oral biofilm-related infections are discussed in the following sections.^4^

## RESISTANCE MECHANISM OF BIOFILM

Bacteria surviving and growing within a biofilm exhibit a tremendous amount of antibiotic resistance. Their stable structural properties and the proximity of the bacterial cells within the biofilm create an excellent environment for horizontal gene transfer, which can lead to the spread of antibiotic-resistant genes amongst the biofilm inhabitants. As per the available literature, the following structural and physiological properties of biofilm-forming bacteria help them become tolerant to antibiotics and slowly become antibiotic-resistant.^4^,^6^

### Capsule

The capsule is an important part of the biofilm; both Gram-positive and Gram-negative bacteria can have capsule thicknesses ranging from 0.2–1.0 μm. Biofilm adhesion and cohesion to a solid surface is by means of the capsule which uses Van der Waal, hydrogen bonds, and electrostatic forces, therefore contributing to the maturation of biofilm. The biofilm capsule consists of polysaccharides and glycoproteins that are influenced by conditions of different environments. The accumulation of antibacterial molecules by the capsule or glycocalyx layer constitutes up to 25% of its weight, resulting in the development of resistance to antibiotics and other antimicrobial drugs by the bacteria. The antimicrobial drugs are trapped in the adsorption sites of the glycocalyx matrix, where these drugs are degraded by the exoenzymes released by the MDR bacteria. Hence, the antimicrobial drugs slowly become ineffective in combating the bacteria present in the biofilm. 4,6,7

### Cell Membrane Modification

Cell membranes are the major target site for major antibiotics; however, alteration or modification of cell membrane permeability and altered expression of porin proteins present in bacteria inside the biofilm do not allow the entry of antibiotics, thus leading to the development of resistance.^4^,^6^,^7^

### Efflux Mechanism

Efflux pumps present in the bacteria are responsible for antimicrobial resistance in biofilm structures. Generally, these efflux pumps are a group of transport proteins that help remove toxic and unwanted substances from the bacteria. These pumps do not allow antibiotics to enter the bacterial cell, leading to the emergence of MDR bacteria.^4^,^7^,^8^

### Plasmids/Enzyme-mediated Resistance

Plasmids are extrachromosomal genetic materials commonly found in biofilm-forming bacteria. Plasmids generally contain genes that are encoded for increased virulence by means of enzymes and proteins, leading to resistance to various antimicrobials and heavy metals. Sometimes these plasmids carry multiple resistant genes that are specifically resistant to most of the commonly used antibiotics, including macrolides, beta-lactams, aminoglycosides and fluoroquinolones. These genes usually get rearranged with the help of an inherent recombination system as seen with integrons and transposons. All these plasmids are conjugative; horizontal transfer of these resistant genes occurs in closely packed oral biofilm. In a slow but confirmative process, slowly all the bacteria become multidrug-resistant.^4^,^7^,^9^

### Genetic Adaptation

Regular exposure to antibiotics and other MDR bacteria results in the direct horizontal transfer of antibiotic-resistant genes and leads to long-term genetic adaptations or mutations in response to an alteration in their growth environment. Such mutations lead to the development of drug-resistant strains.^7^,^9^

### Quorum Sensing

Several bacteria regulate their metabolic/physiological activities through a mechanism called quorum sensing, where the individual bacteria communicate with each other by releasing diffusible components. Quorum sensing facilitates interactions among bacteria, which leads to host colonization, the formation of biofilms, and their defense against antimicrobials, the host defense system, and their adaptation to an ever-changing environment. Clearly, a degree of quorum sensing in pathogenic bacteria is responsible for their virulence and breaking down the host defenses.^4^,^10^

### Metabolic Factors

Differences in oxygen availability and nutrients inside the biofilm affect the metabolic action and growth rate of the bacteria. Varying concentrations of metabolic substrate and by-products affect the growth of bacteria within the biofilm, which leads to heterogeneity in the growth of the microbial population. The available nutrients and fluctuating concentration of oxygen affect the metabolic activities of cells in the peripheral region of biofilm that supports the proliferation of different species of bacteria. The metabolically active bacteria are killed by biocides, whereas the dormant bacteria which are less vulnerable to the antimicrobial drugs survive for a long period.^7^,^10^

### Persistence Bacteria

The antimicrobial agent-tolerant cells, called persisters, are responsible for severe chronic infections. The major challenge in most dental clinics is the detection of bacterial strains. The lowering of the ATP level decreases antibiotic target activity, leading to the formation of persisters. The resistant persister cells in the bacterial biofilm exhibit antibiotics tolerance.^4^,^11^

### Stress Response

The stress response in a biofilm is attributed to the change in the structural and physiological functions of bacteria that lead to an increase in their stress tolerance capacity. Normally, the formation of the cell envelope and synthesis of thin aggregative fimbriae are controlled by a stress response in *Salmonella enteritis* and *Escherichia coli*. The stress response also helps in their cellular damage repair mechanism. Stress may be induced for several reasons, including deprivation of nutrients caused by the stationary phase in the growth of bacteria, at high or low temperatures, acidic pH, and higher osmolality.^4^,^11^

## MANAGEMENT OF ORAL BIOFILM

A number of different articles in the scientific literature describe the following techniques for effective management and removal of oral biofilms.^12^–^19^

### Antimicrobial Material

The use of antimicrobial materials is vital to prevent bacterial adhesion and formation of a biofilm, as most infections in the oral cavity originate from it. Extensive efforts have been made to provide dental materials with antimicrobial properties, aimed antimicrobial agent release, contact killing, and multifunction.^12^

### Antiplaque/Antimicrobials

Antiplaque agents such as essential oils (eugenol, clove oil) and surfactants (sodium lauryl sulphate) have been effective in removing dental plaque. Antimicrobial agents such as bisbiguanides, metal ions, phenols, and quaternary ammonium compounds have been successfully formulated along with the addition of antiplaque agents into toothpaste and mouthwash for control and removal of oral biofilms/dental plaque.^12^–^14^

### Antimicrobial Release Agent

A very specific technique is used to kill the bacteria inside the biofilm; antimicrobial agents are preloaded and incorporated in the dental materials used for various dental treatments. Antibiotics, in a combination of silver compounds, were the first kind of antimicrobial agents used for biofilm removal. The main advantage of this technique is that even at very low concentrations, antimicrobial agents against biofilms are released to prevent bacterial growth. In addition, using a antimicrobial release agent provides a high local dose at the site of interest, strong broad-spectrum antimicrobial activity, reduced systemic toxicity, and minimal risk of antimicrobial resistance. However, this technique also has some disadvantages: it is short-acting, and exhaustion of the antimicrobial reservoir in the dental material can occur. Currently, nanotechnology is being used to incorporate various antimicrobial agents into dental materials, providing a better antimicrobial effect, and for a longer duration.^12^

### Contact-killing

The contact-killing strategy is based on the strategic incorporation of a wide range of antimicrobial agents (synthetic chemicals such as quaternary ammonium compounds and polycations to natural biomolecules like antimicrobial peptides) into dental materials. The compounds are non-toxic and can be easily mixed with dental materials to form a covalent compound structure. These are broad-spectrum antimicrobial agents that have a strong effect on both Gram-positive and Gram-negative bacteria when they both come in contact with the dental material. This strategy has several advantages, being non-toxic, with long-term anti-microbial activity and non-irritant properties, while drawbacks include the fact that it has a bacteriostatic instead of a bactericidal effect, and that surface biofouling occurs (roughness on the surface of the teeth).^12^

### Multifunctional Mechanisms

Antimicrobials with multiple or broad-spectrum activities have been preferred over the years for increasing the efficacy of controlling oral infections caused by various bacteria, viruses, and fungi. Silver compounds with free silver, silver nanoparticles (AgNPs), have a high surface-to-mass ratio, and the control of releasing kinetics is easier, as is the long-term maintenance of the antibacterial effect. In experimental dental adhesives and composites, very low concentrations of AgNPs are effective against plaque biofilms. The combined action of AgNPs and quaternary ammonium salts to enhance the antibacterial effects has been recently adopted for the preparation of antimicrobial dental materials. Antimicrobial dental material provides effective killing of bacteria on its own surface, as well as away from it. While such materials are multifunctional, there are currently not enough combinations available that provide synergistic and enhanced antimicrobial properties, and more research is needed.^12^

### Nanoparticles

Removal of oral biofilm using nanoparticles has enormous potential. Nanoparticles have excellent antibacterial activity and can be used to target specific biofilm-forming microorganisms without disturbing the normal microflora of the oral cavity. However, their use is expensive and cannot be regularly practiced in dental clinics. Administering nanoparticles requires great precision and may lead to severe side effects. Furthermore, the biomimetic properties of nanoparticles should be highly precise and target-specific to achieve the desired results.^15^

### Photodynamic Therapy

Photodynamic therapy (PDT) is an emerging non-invasive technique that is employed against oral infections, especially periodontal infections. Photodynamic therapy involves an oxygen-dependent photochemical reaction that occurs upon light-mediated activation of a photosensitizing compound leading to the generation of cytotoxic reactive oxygen species, predominantly singlet oxygen. The therapy is directly applied topically to the periodontal pockets, together with an antimicrobial agent for the removal or killing of the microorganisms. Factors such as overdose and exposure duration should be always taken into consideration to minimize side effects and retain the normal microflora. Photodynamic therapy also minimizes the emergence of MDR strains.^12^,^16^

### Cold Atmospheric Plasma

Cold atmospheric plasma (CAP) is a non-invasive technique used in dentistry and oncology for its antimicrobial and cell necrosis properties. Non-thermal plasma at a temperature of less than 104°F is administered at the application site. This method uses a highly reactive mix of ions and electrons, radical species, molecules in the ground or excited state, and quanta of electromagnetic radiation (UV photons and visible light). Treatment with CAP is carried out under normal atmospheric conditions, which makes it easier for *in vivo* applications without destroying the surrounding tissues and microflora. Apart from the removal of dental plaque, CAP has been used in bleaching, endodontic treatment, sterilization of surgical instruments, and composite restoration. However, more studies are needed to assess the ease of CAP usage and to increase its field of application.^12^,^17^

### Herbal/Natural Products

Plants such as *Salvadora persica*, *Allium sativum*, *Punica granatum*, as well as naturally occurring compound honey have been reported for their biofilm removal properties. One of the most important aspects of herbal products or naturally occurring compounds is their complex chemical structure, for which bacteria fail to develop resistance. However, their therapeutic action can be slow, and sometimes the ideal dosage versus toxicity may become a concern. Nevertheless, they can serve as an excellent adjunct in combination with antibiotics, or other antimicrobials, with a good synergistic effect. Hence, more natural and herbal compounds should be explored for the treatment of oral biofilms.^18^,^19^

## CHALLENGES FACED IN ORAL BIOFILM REMOVAL

There are consequences to the global widespread use of antibiotics at variable concentrations. Such use may not only affect the microbiota; variable genetic effects can also occur, such as antimicrobial resistance, a mutation that has a direct effect on humankind. Furthermore, biofilms are already highly defensive and resistant to the action of antibiotics.^20^ The surface-associated infections, which increase by the colonization of bacteria and physiological alteration within the biofilm, significantly escalate the problem.^20^ The major hurdles in treating oral biofilms are the infections that arise through the devices used in patients during the treatment process. 21–24 The resistance of bacteria in oral biofilm to antibiotics is a longstanding problem in patient management. In addition, MDR bacteria in the biofilm are composed of both Gram-positive and Gram-negative bacteria, thereby providing a high chance of recurrence. Several ongoing lines of research have demonstrated the presence and transfer of drug resistance genes and proteins within other normal flora and biofilm MDR-forming bacteria.^25^ Management of oral biofilms requires much more investigation, which will help in the understanding of the complex interaction between the host defense system and biofilm communities.

## CONCLUSION

Several therapeutic approaches have been utilized over the years for the effective management of oral biofilms and related infections. However, the development of MDR bacteria has made many of these procedures ineffective. Moreover, some of these procedures are also detrimental to the normal flora present in the oral cavity. In light of this, it is very important to use the correct combination of treatment procedures and materials, which are not only more target-oriented, but also have fewer side effects.

## Abbreviations

CAP: cold atmospheric plasma
EPS: extracellular polymeric substance
MDR: multidrug-resistant
PDT: photodynamic therapy
QS: quorum sensing
